# Environmental Effects on Strength and Failure Strain Distributions of Sheep Wool Fibers

**DOI:** 10.3390/polym14132651

**Published:** 2022-06-29

**Authors:** Olesja Starkova, Alisa Sabalina, Vanda Voikiva, Agnese Osite

**Affiliations:** 1 Institute for Mechanics of Materials, University of Latvia, Jelgavas 3, LV-1004 Riga, Latvia; alisasabalina@gmail.com; 2 Institute of Chemical Physics, University of Latvia, Jelgavas 1, LV-1004 Riga, Latvia; vanda.voikiva@lu.lv; 3 Department of Analytical Chemistry, University of Latvia, Jelgavas 1, LV-1004 Riga, Latvia; agnese.osite@lu.lv

**Keywords:** single fiber test, tensile properties, Weibull distribution, gauge length, environmental degradation, UV aging, moisture, durability

## Abstract

Sheep wool is an eco-friendly, renewable, and totally recyclable material increasingly used in textiles, filters, insulation, and building materials. Recently, wool fibers have become good alternatives for reinforcement of polymer composites and filaments for 3D printing. Wool fibers are susceptible to environmental degradation that could shorten their lifetime and limit applications. This study reports on the mechanical properties of sheep wool fibers under the impact of humid air and UV irradiation. The results of single fiber tensile tests showed a noticeable gauge length effect on the fibers’ strength and failure strain. Long (50 mm) fibers possessed about 40% lower characteristics than short (10 mm) fibers. Environmental aging decreased the elastic modulus and strength of the fibers. Moisture-saturated fibers possessed up to 43% lower characteristics, while UV aging resulted in up to a twofold reduction of the strength. The most severe degradation effect is observed under the coupled influence of UVs and moisture. The two-parameter Weibull distribution was applied for the fiber strength and failure strain statistical assessment. The model well predicted the gauge length effects. Moisture-saturated and UV-aged fibers were characterized by less extensive strength dependences on the fiber length. The strength and failure strain distributions of aged fibers were horizontally shifted to lower values. The results will contribute to be reliable predictions of the environmental durability of sheep wool fibers and will extend their use in technical applications.

## 1. Introduction

Increased concerns on availability, climate neutrality, and sustainability of available resources set new standards for the design of novel eco-friendly materials, their practical use, and planning of the end-of-life of products. Owing to their abundance and biodegradability, natural fibers have become good candidates for substituting for fossil-based counterparts in some technical applications [1,2,3].

Animal fibers are the second most widely used natural fibers after plant fibers (e.g., flax, cotton, hemp, jute, kenaf, abaca, etc.). Sheep wool is the most used commercially, particularly in the apparel and textile industry, agriculture, filtering elements, and thermal and acoustic insulation materials [3,4,5,6]. For instance, around 450 tons of wool are produced annually in Latvia [7]. According to rough estimates, only about 30% of the collected wool is used to make yarn, while the rest of the wool mostly becomes an agricultural waste [8]. Along with its availability, cost-effectiveness, biodegradability, and sustainability, wool has a range of unique properties that can expand applications of this natural material to other sectors and convert waste to resources. High hygroscopicity, thermal and sound insulation, flame retardant, and antistatic properties are desirable for materials used in building and automotive sectors [9,10]. Wool fibers are durable and flexible; their mechanical characteristics are comparable to or exceed the values of other natural fibers, e.g., cotton and coir/husk [1,11]. Thus, wool is used as fiber reinforcement in mortars and concrete [9,12,13], polymer composites, and laminates [2,14,15,16,17], increasing the value of the wool fiber. Recently, wool has become an alternative material in some high-tech industrial sectors, e.g., it is used in reinforcing compounds for additive manufacturing [11,18].

Wool is the natural protein fiber obtained from sheep, goats, camels, rabbits, and other mammals. It comprises hair-like multicellular fibers which grow out of skin follicles at a rate of around 100 mm per year [9]. Wool is mainly composed of keratin; the fibers have a complex structure and consist of a cortex and a surrounding cuticle layer [5]. A scaly fiber surface makes it easier to spin the fleece, which is used in textile fabrics and contributes to enhanced adhesion, which is favorable in reinforcing polymer compounds. The structure and mechanical properties of wool fibers depend on the breed, sex, and age of the sheep [19,20]. Bouagga et al. showed that variations in the elastic modulus and strength of the Tunisian sheep wool fibers of different breeds and sexes are in the range of 5–20%, and they correlated these data with fiber diameters, crystallinity degree, and cysteine amount [19].

Like many natural fibers, wool fibers possess a complex internal structure and marked variability in geometrical characteristics and mechanical properties [21,22,23]. Unlike most man-made fibers, which are geometrically uniform, wool exhibits between-fiber and within-fiber diameter variations caused by, for example, changing growing conditions. Fiber flaws or morphological defects, which can be built-in or induced during processing, serve as initiation sites of fiber failure. The strength of natural fibers exhibits a substantial scatter on diameter and dependence on fiber length [23,24,25]. In the study of Guo et al. [25], the strength of short (10 mm) palm fibers is 20% higher than that of long (40 mm) fibers. Sia et al. [23] reported strength variations up to 16% for banana fibers of different lengths. Parlato et al. [13] studied low-quality wool of a Sicilian sheep breed and reported on strength decrease within the fiber diameter: from 200 MPa for 50 μm fibers down to 50 MPa for 90 μm fibers.

According to the weakest link concept, fiber fails at a point with an internal flaw or where fiber diameter is small, or a combination of both. The amount of such “weak links” and the probability of reaching their breaking limits increases with the growing length of fibers. Thus, longer fibers are typically characterized by lower strength and greater data scatter [22]. Due to the wide dispersion of mechanical properties, statistical analysis methods are required to evaluate the fibers’ probabilistic strength and failure strain. Weibull distribution is commonly utilized to study the discrete fiber property [21,26,27]. The two-parameter Weibull distribution is among the most used models for assessing natural fibers’ strength scatter characteristics [22,23,24,25]. For fibers possessing essential diameter variations, the modified Weibull model is applied to obtain accurate predictions [13,22,25].

Fibers of natural origin are generally susceptible to environmental degradation. This fact can essentially reduce the durability of natural fiber-based products and complicate the prediction of their long-term performance [28]. Heat, UV light, and atmospheric humidity are among common external factors affecting fiber appearance and performance during their growth, processing, and use. Wool fibers composed of keratin are more susceptible to chemical damage and unfavorable environmental conditions than the cellulose in the plant fibers [2]. Thermal and UV aging destroy wool fibers’ surface composition, leading to their yellowing and up to a twofold reduction of their strength [29]. Wool is hydrophilic and absorbs water up to 30% of its weight [2,11]. Absorbed moisture plasticizes the fibers, increasing their stretching ability while reducing the elastic and strength characteristics [9,13]. In order to fully characterize the mechanisms of environmental impact on fibers, it is necessary to evaluate not only the actual change in their properties but also the change in the distribution parameters depending on fiber geometrical characteristics.

Most of the works reported in the literature focused on the characterization of specific sheep wool fibers and determining their strength, and rarely on failure strain and distribution [22,30,31]. A few works highlighted environmental impacts on the mechanical characteristics of sheep wool [9,13] and some plant fibers [26]. However, no systematic studies on the analysis of the strength and failure strain distributions of aged fibers with their length variations were found in the literature. 

The present study is aimed to evaluate the strength and failure strain distributions of the sheep wool fibers under the effect of environmental factors. The single and coupled influence of moisture and UV irradiation on tensile properties of Latvian dark-headed sheep wool fibers is studied on fibers of different lengths. The total experimental campaign on around 300 virgin and environmentally aged fibers was carried out, and the results were statistically analyzed using Weibull distribution. The dependences of the strength and failure strain on the gauge length of fibers exposed to different environments were obtained and fitted by the two-parameter Weibull model. The obtained results contribute to the comprehension of the environmental durability of products made of wool fibers and reliable predictions of their long-term performance. This, in turn, will promote local development opportunities and waste management through extended use of natural and renewable local resources for the development of novel products of low carbon impact and energy-efficient properties.

## 2. Materials and Methods

### 2.1. Wool Fibers

**Figure 1:**
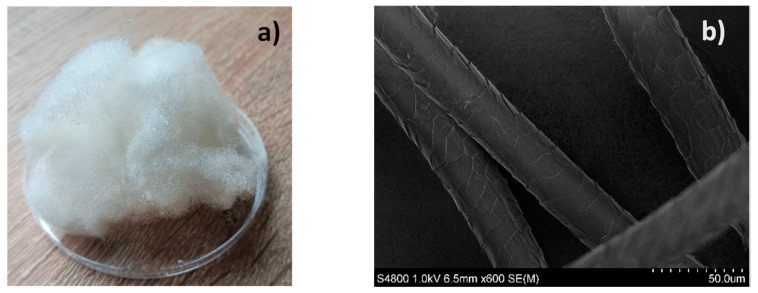
Photo of a wool ball (**a**) and individual fibers by SEM (**b**).

The wool samples were collected according to Australian Standard AS/NZ 4492.1 from the Latvian dark-headed breed sheep of a similar age (2–4 years) and the same gender (female). Animals of this age and gender were chosen because they make up the majority of sheep flocks not only in Latvia but throughout the world. The wool is sheared regularly, which makes it more homogenous. Thus, these samples are more representative of the characteristics of wool fibers. The collected sheep wool fibers were washed with detergent (soap with surfactants) in warm water (50–60 °C) and rinsed several times in water. Then they were dried at room temperature, in air, for a day [6]. A photo of an original wool ball and an SEM micrograph of individual fibers are shown in Figure 1. Single fibers for testing were carefully pulled out of the woolball one by one.

### 2.2. Single Fiber Tests

**Figure 2:**
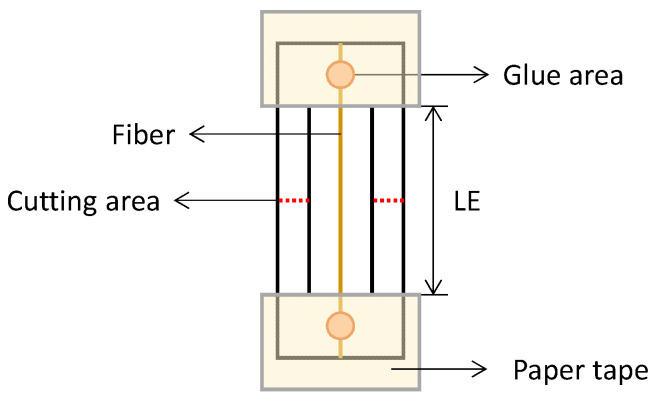
Schematic drawing of a fiber mounted in a paper frame for a tensile test.

Quasistatic tensile tests were made according to ASTM D3379-75 by using the universal testing machine, Zwick, with a 100 N load cell. The wool fiber was mounted on a paper frame and additionally fixed with adhesive paper tape tabs (Figure 2). Fibers of different gauge lengths (LE) were studied: LE = 10, 30, and 50 mm (denoted in the text as LE10, LE30, and LE50, respectively). Scissor cuts were made on both sides of the paper frame tabs at the mid-gage just before the start of the test. The tests were performed at the crosshead speed rate of 1 mm/min under ambient conditions (22 ± 1 °C, RH = 37 ± 5%). The diameter of each fiber was measured by an optical microscope (equipped with a Moticam 2300 digital camera) with 8X magnification at five points along its length, and the average value was used to calculate the cross-sectional area. Between 20 and 40 tests were completed for each specific group of fibers conditioned under different environments, with different LE (Table 1).

### 2.3. Fiber Conditioning

Fibers mounted in paper frames were conditioned under different environments (Table 1). “As produced”, samples were stored in a plastic box under ambient conditions (relative humidity RH = 37 ± 5% and temperature *T* = 22 ± 2 °C). These fibers were considered as the reference samples. A part of the reference fibers was placed in a desiccator under a saturated salt solution of K_2_SO_4_, giving an RH = 98%, under an ambient temperature. This group of samples was divided into two depending on the duration of fiber exposure in this environment: 1 week and 2 months for “RH98” and “RH98-ext”, respectively (“ext” means extended time). Another part of the reference fibers was exposed to ultraviolet irradiation (denoted as “UV”) for 24 h. Irradiation tests were made by using a high-pressure mercury-vapor discharge lamp, UV DRT230, as a UV source, giving a UV-A emission with the strongest peak at about 365 nm. The irradiation intensity was adjusted to 4 mW/cm^2^. The temperature under the lamp was maintained at 35 ± 2 °C. In addition, UV irradiation effects were studied on fibers preliminary conditioned under a humid environment. This group of fibers is denoted as “UV-RH98”.

### 2.4. Scanning Electron Microscopy (SEM)

SEM images were obtained using a Hitachi S4800 Scanning electron microscope, with an operating voltage of 1.0 current kV, with 5–7 A. Wool fibers samples were electrically bonded to a sample analysis table with electrically conductive tape, and measurements were made. Reference and UV fibers were studied.

### 2.5. Optical Microscopy

Optical micrographs were taken using an inverted microscope (Olympus IX 71) in the PL mode using an Hg lamp light source (U-LH100HG) with a fluorescence filter set (U-MWU2), and in the micro-extinction spectroscopy (MExS) transmission mode, with a 10× objective lens (CPLNFLN 10XPH, NA 0.3).

## 3. Statistical Analysis with Weibull Distribution

Weibull distribution based on the weakest link theory for the failure strength, σ, states that the probability of failure P(σ) of a material component of volume V is [25,32,33].

(1) $$P\left(\sigma \right)=1-\exp\left[-\frac{V}{V_{0}}{\left(\frac{\sigma }{\sigma_{0}}\right)}^{m}\right]$$

where $V_{0}$ is the unit volume, *m* is the shape parameter (Weibull modulus), and $\sigma_{0}$ is the characteristic strength or scale parameter. The strength distribution with a lower *m* tends to perform a larger scatter and vice versa. Equation (1) is the most widely used formulation of Weibull distribution, called a two-parameter Weibull distribution function, used in failure analysis of various fibers.

The value *P* is estimated using a probability index, e.g.,

(2) $$P=\frac{i-0.3}{N+0.4}$$

where *i* is the rank of the respective data points of strength placed in ascending order, and *N* is the total number of data points (tests/fibers). Alternative probability indices are given in [33].

When the cross-sectional area of all fibers is the same, the volume, *V*, in Equation (1) can be replaced by the gauge length, *L*. In a general case of fibers with geometrical irregularities, Equation (1) can be rewritten in the following form [13,21,22]:

(3) $$P\left(\sigma \right)=1-\exp\left[-{\left(\frac{L}{L_{0}}\right)}^{\alpha }{\left(\frac{\sigma }{\sigma_{0}}\right)}^{m}\right]$$

where *L* is the gauge length of the fiber and *L*_0_ is the unit length (normally *L*_0_ = 1 for mathematical convenience). Parameter α (0 < α ≤ 1), known as the Gutans–Tamuzs [34] or Watson–Smith [35] parameter, is introduced in order to account for diameter variations [13,22,23]; α = 1 for a constant within-fiber diameter.

Rearranging Equation (3) while taking the logarithm of both sides provides the following formula:

(4) $$\ln\left(-\ln\left(1-P\right)\right)-\alpha \;\ln\left(L/L_{0}\right)=m\;\ln\sigma -m\;\ln\sigma_{0}$$

As follows from Equation (4), by plotting $\ln\left(-\ln\left(1-P\right)\right)-\alpha \;\ln\left(L/L_{0}\right)$ against lnσ, a linear graph is produced. Parameter *m* represents the slope of this line, while *σ*_0_ is estimated from the intercept with the ordinate for the given *L* and α. The parameter α in Equations (3) and (4) is determined using the coefficient of variation of the diameter $CV_{d}$ (i.e., the average within-fiber diameter variation of *N* fibers), and α represents the slope of $\ln\left(CV_{d}\right)$ versus ln(L) line [22,23,25,32]. The diameter variation among fibers can be neglected for long samples since their average diameter at each gauge length is close to each other [22].

Once the Weibull distribution parameters (*m*, $\sigma_{0}$) and diameter variation parameter, α, are determined, the average value of the strength is obtained [24,25]

(5) $$\langle \sigma \rangle =\sigma_{0}{\left(\frac{L}{L_{0}}\right)}^{-\frac{\alpha }{m}}\Gamma \left(1+\frac{1}{m}\right)$$

where Γ(*x*) is the gamma function.

Analogously to the failure strength distribution in Equation (3), the two-parameter failure strain, *ε*, distribution can be written as [21,22]:

(6) $$P\left(\varepsilon \right)=1-\exp\left[-{\left(\frac{L}{L_{0}}\right)}^{\alpha }{\left(\frac{\varepsilon }{\varepsilon_{0}}\right)}^{m_{\varepsilon }}\right]$$

where $m_{\varepsilon }$ and $\varepsilon_{0}$ are the shape and scale parameters, respectively.

The mean failure strain is determined by the relationship similar to Equation (5):

(7) $$\langle \varepsilon \rangle =\varepsilon_{0}{\left(\frac{L}{L_{0}}\right)}^{-\frac{\alpha }{m_{\varepsilon }}}\Gamma \left(1+\frac{1}{m_{\varepsilon }}\right)$$

A procedure for the determination of $m_{\varepsilon }$ and $\varepsilon_{0}$ is analogous to this described above for m and *σ*_0_.

## 4. Results and Discussions

### 4.1. Diameter Variations

**Figure 3:**
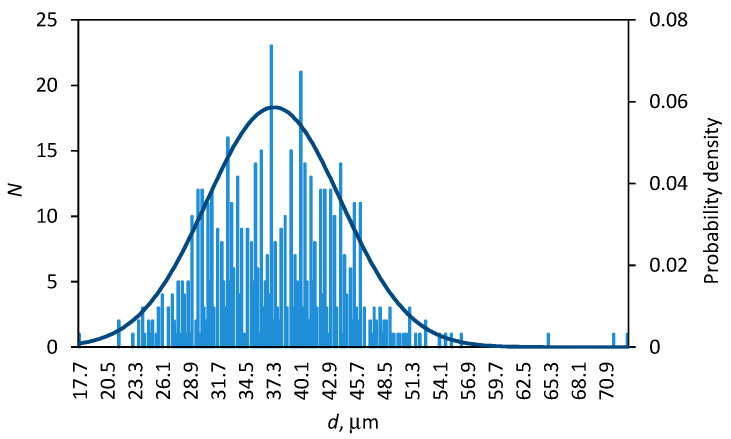
Distribution of the reference wool fiber diameters; the line is the probability density by a normal distribution.

Wool fibers are traditionally positioned as fibers with geometrical irregularities for which diameters vary greatly, not only among fibers but also along the fiber length [22]. The distribution of the diameters measured for the whole population of the reference wool fibers (total of 625 measurements) is shown in Figure 3. As seen from the histogram, diameter values lie in the range of 17–73 μm with the mean value of 37.4 (±6.8) μm. These values are in accordance with literature data for other sheep wool fibers: 15–50 μm [11], 50–90 μm [13], and 25 μm [22].

**Figure 4:**
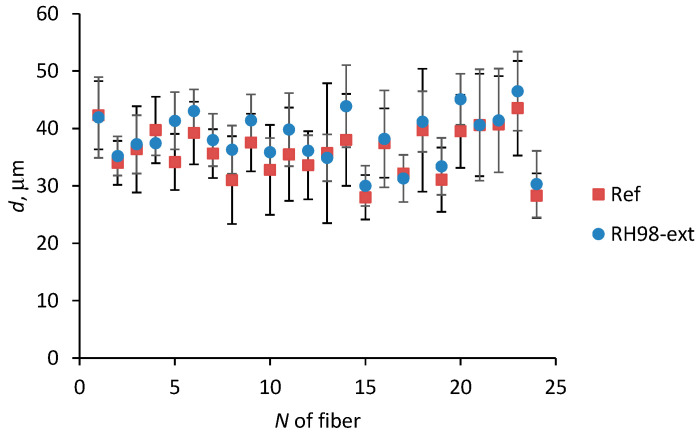
Diameter of fibers in the reference state and after exposure under an RH of 98%.

After exposure of fibers to a humid environment and UV rays, their diameters did not change significantly, and *d* values remained within the distribution for the reference samples. The mean values of diameters for fibers of different gauge lengths in the reference and aged states are listed in Table 2. Figure 4 demonstrates an example of diameter variations of 24 different fibers due to their conditioning in a humid environment (“RH98-ext” samples, Table 1). Each point on the graph is the average from 5 measurements within the same fiber before and after its aging.

### 4.2. Tensile Properties of Wool Fibers

**Figure 5:**
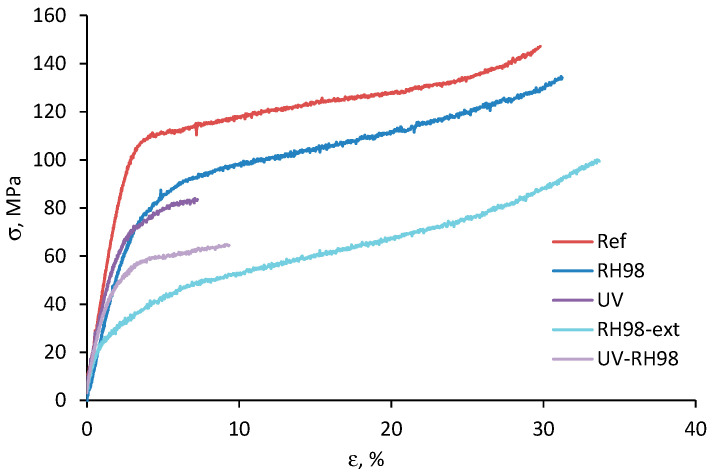
Representative stress-strain diagrams of wool fibers exposed to different environments; LE = 30 mm.

Representative stress-strain diagrams of the reference and aged fibers are shown in Figure 5. The wool fibers possess a highly non-linear viscoelastic–viscoplastic behavior typical for many natural fibers [13,21,22,25,26]. Generally, four regions can be distinguished on the stress-strain curve: (i) an almost linear part at low strains below 2–3%; (ii) a non-linear region with decreasing stress gradient ending with a yield; (iii) a steady-state phase with a constant stress gradient; (iv) a stress hardening region up to fiber breakage. The last phase is not always present, and failure occurs before the final stage (e.g., for UV-irradiated fibers). The elastic modulus was determined in the linear part of the stress-strain curve, i.e., in the region I.

The average values of the elastic modulus (*E*), strength (*σ*), and failure strain (*ε*) for all the groups of wool fibers are listed in Table 2. The reference fibers of a 30 mm gauge length are characterized by <*E*> = 3.93 ± 0.61 GPa, <*σ*> = 142.8 ± 30.3 MPa, and <*ε*> = 25.9 ± 11.4%. These data compare well to those reported in the literature for other types of sheep wool [1,2,9,13,18], although they are somewhat smaller than those obtained with high-quality Merino wool [22] and some other natural fibers [1,11,36]. The specific strength and stiffness of the sheep wool fibers are comparable with some wood and plant fibers and synthetic polymer fibers. These values confirm the suitability of wool fibers as a reinforcement material for concrete and polymer composites [3,5,13].

The presented data (Table 2) reveal the gauge length effect on the fiber strength that is compatible with the weakest link concept. The strength and failure strain of long (50 mm) fibers are about 40% lower than those for short (10 mm) fibers. A larger gauge length indicates a higher probability of defects, larger flaw numbers, and, thus, lower tensile strength [21,25]. Like all natural fibers, wool fibers inevitably develop internal defects during their growth. Fibers break in the defective or weakest parts; thus, each strength value in tests represents the strength of the weakest part of each fiber [24]. The strength of fibers reduces as the gauge length increases due to the increased number of flaws that appeared at a longer length. For instance, Zhang et al. [22] reported on the gauge length effect on the strength of merino wool fibers: 215 MPa for short (10 mm) and 200 Mpa for 100 mm long fibers. Similar effects are observed for various plant fibers [21,23,25,33].

Environmental aging significantly affected the mechanical performance of the wool fibers. Aged fibers possessed lower strength and stiffness compared to their pristine counterparts (Figure 5). Stretching ability drastically decreased for UV-aged fibers, while remaining unchanged or increased for moisture-saturated samples. At the same time, UV irradiation moderately affected the elastic modulus of the fibers. According to the data in Table 2 and comparing the average values for the reference and aged fibers of a 30 mm gauge length, the strength reduction is by 13%, 34%, 42%, and 53% for RH98, RH98-ext, UV, and UV-RH98 samples, respectively. For comparison with other studies, the strength of dry and wet (100% RH) wool fibers differ by about 16% (260 MPa and 190 MPa, respectively) [9] and 12% (86 MPa and 75 MPa) [13]. Similarly, the elastic modulus decreased by 22%, 43%, and 25% for RH98, RH98-ext, and UV-RH98 samples, respectively. It can be concluded that the combined action of moisture and the UV resulted in higher fiber degradation compared to that of single environmental factors. Long-term (2 months) conditioning under a humid environment affected the strength and elastic modulus of the fibers to a greater extent than their 1-week exposure. This fact cannot be solely related to the plasticization effect of absorbed moisture, but it is associated with moisture-induced structural degradation. The latter can, in turn, result in an additional moisture ingress into the fibers and increase the negative impact on the strength. According to the data of our previous study on moisture diffusion into the sheep wool (not shown here), the “Fickian” saturation was achieved in 3 days and reached the value of about 23%. Thus, RH98 fibers were assumed as fully saturated. In another study [13], it is reported that ten minutes is a sufficient time for wool fibers to reach saturation in distilled water. The moisture content of RH98-ext fibers was not evaluated in this study. At the same time, it should be noted that neither short-term nor long-term exposure of the fibers to a humid environment resulted in a noticeable change in their diameter (Section 4.1) and visual appearance (Section 4.5). It is assumed that two counterbalancing processes are taking place in moisture-saturated fibers. Absorbed moisture plays a role of a plasticizer and facilitates movements of macromolecular chain segments of keratin, the main structural part of sheep wool fibers. This results in increased failure strains. At the same time, water ingress results in swelling and hydrolytic degradation, leading to the development of additional defects in the fiber structure. Then, an opposite effect and decrease of the ultimate strains are expected.

**Figure 6:**
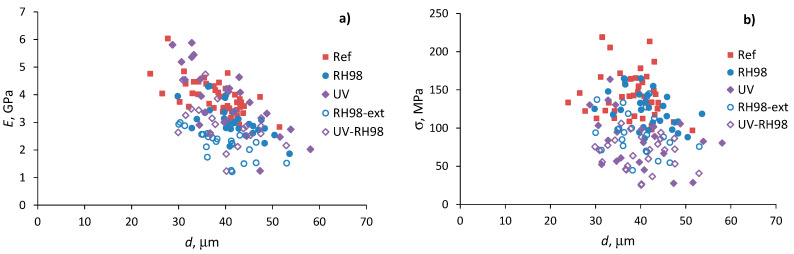
Elastic modulus (**a**) and strength (**b**) as functions of the diameter of the reference and aged wool fibers; LE = 30 mm.

The mechanical properties of natural fibers are well known to be strongly dependent on their diameter [13,21,24]. Figure 6 demonstrates the elastic modulus and strength as functions of diameters of the reference and aged wool fibers of the gauge length LE = 30 mm. Each point on the graphs corresponds to the data from one test of a fiber with an average within-fiber diameter, *d*. A decreasing trend is observed for the elastic modulus: fibers of greater diameters possess lower stiffness in each sample group (Figure 6a). Similar trends were reported for flax [21] and “Valle del Belice” sheep wool [13] fibers. Strength exhibits high variability from fiber to fiber, although with no clear trend on their diameter (Figure 6b). Environmental aging decreased the mechanical characteristics of the fibers but did not affect the overall trends of their changes with diameter variations.

**Figure 7:**
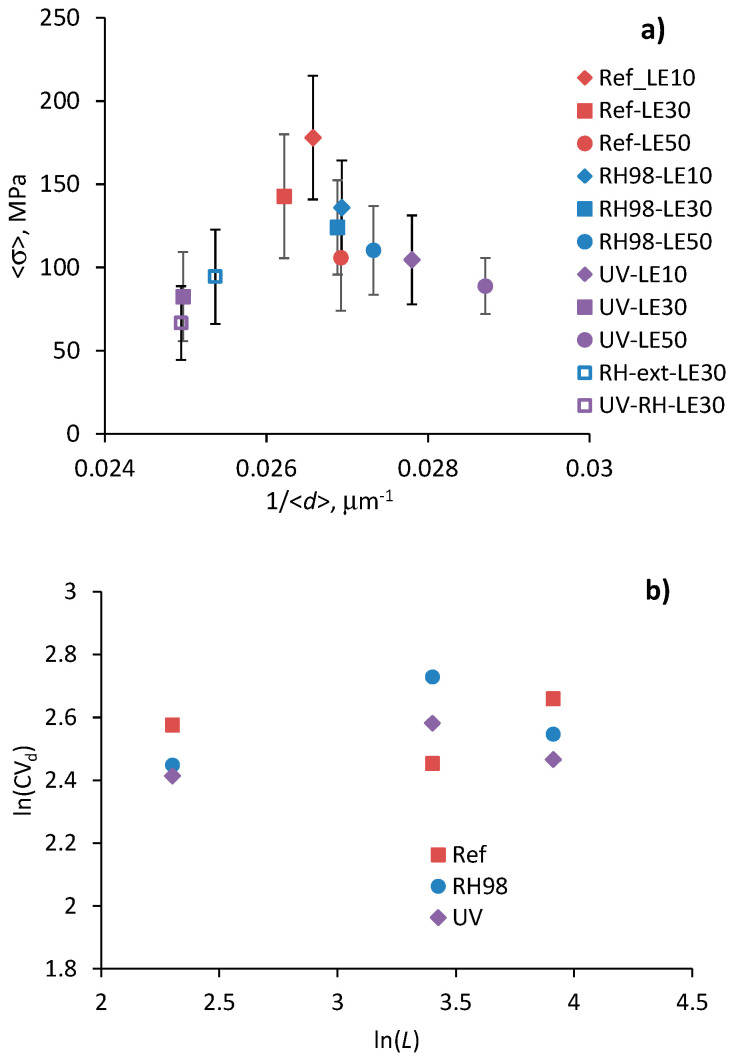
Average fiber strength as a function of fiber diameter (**a**) and relationship between the coefficient of variation of diameter and fiber length (**b**).

According to literature data, the strength of fibers is assumed to be inversely proportional to their diameter [24]. However, this is not the case for the wool fibers under study. Figure 7a demonstrates the average strength, <*σ*>, as a function of the average fiber diameter, <*d*>, for all groups of samples. The strength values are within the data scatter range and do not indicate any specific relationship between <*σ*> and <*d*>. It is also known from the literature that the variability of diameters, expressed through the coefficient of variation $CV_{d}$, increases with the length of fibers [22,23]. The linear log–log dependence between these two parameters gives the parameter α in Equation (3), which represents the slope of the line [25,32]. Figure 7b shows ln($CV_{d}$) versus ln(*L*) dependence for the wool fibers under study. Contrary to the premises, no clear trend between these two parameters is observed. Thus, effects from diameter variation among fibers are ignored in further data analysis, and α = 1 in Equations (3)–(7).

**Figure 8:**
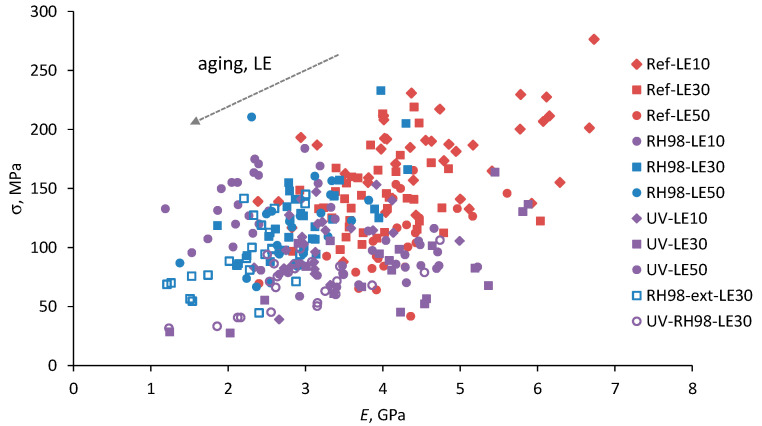
Strength versus the elastic modulus for wool fibers of different lengths (LE) from different environments.

The axial strength and stiffness of fibers are determined by their internal structure; thus, these mechanical characteristics are usually correlated [18,21]. In addition, this relationship, although with some deviations, remains valid after the environmental aging of a material [37]. Figure 8 shows the strength versus the elastic modulus for all tested samples, i.e., for the reference and aged wool fibers of different gauge lengths. One point on the graph represents the data of a tensile test of a specific fiber. Despite noticeable data scatter, a definite trend is observed: fibers with a higher elastic modulus possess higher strength. Environmental aging results in a decrease of both mechanical characteristics. Longer fibers possess lower *σ* and *E* values within each group of aged samples (Figure 8 and Table 2).

### 4.3. Weibull Strength Distribution Analysis

**Figure 9:**
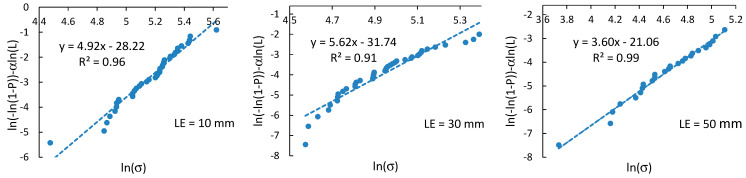
Weibull plots for fiber strength at different gauge lengths.

Based on Equation (4), modified Weibull linear plots of ln(−ln(1−P))−αln(L) vs. lnσ of the reference fibers at different gauge lengths are shown in Figure 9. Similar plots were obtained for environmentally aged fibers. Due to weak correlations between the ultimate properties and diameter of fibers (Figure 7), α was assumed to be equal to unity in all calculations. The correlation coefficients, *R*^2^, are mostly in the range of 95%–98% (Table 2), indicating a reasonable degree of linearity between the linear regression of the fiber strength and experimental data.

**Figure 10:**
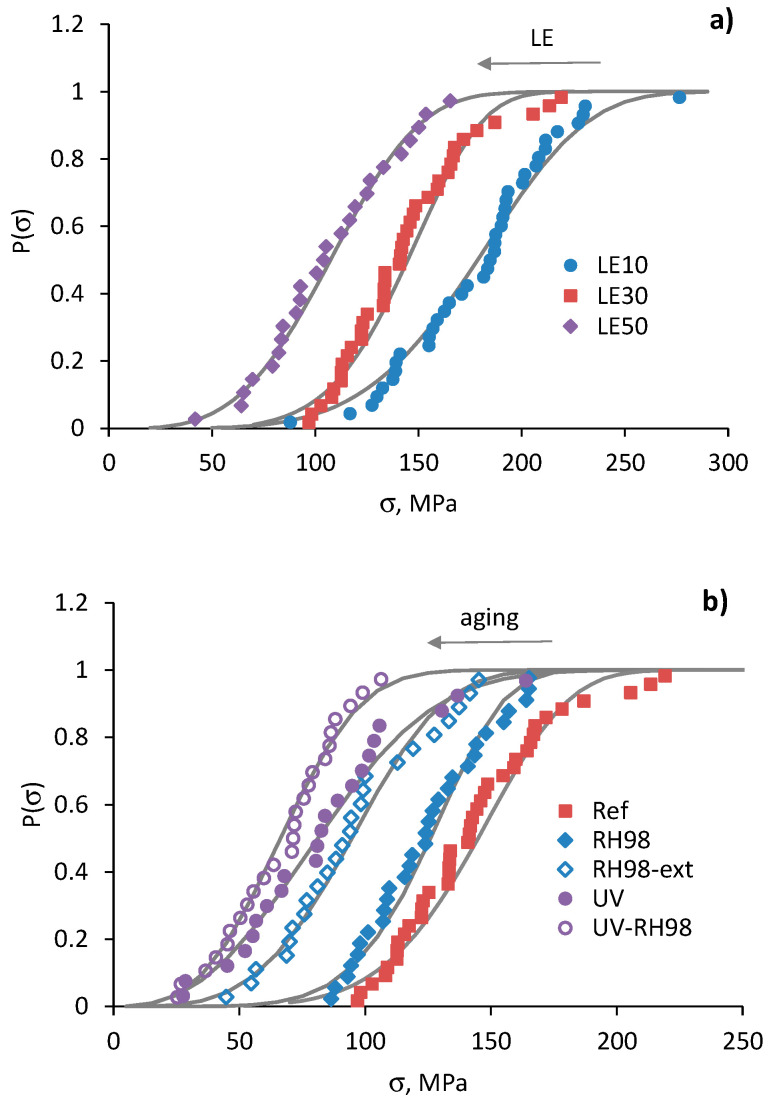
Strength distribution of the reference fibers of different gauge lengths (**a**) and fibers from different environments with LE = 30 mm (**b**). Lines are calculations by Equation (3).

The strength distribution of the reference and environmentally aged fibers is shown in Figure 10. The data confirm the applicability of the Weibull distribution for the wool fiber tensile strength analysis both in the reference state and after their aging. An increase in the gauge length of fibers, as well as their exposure to a humid environment and UV irradiation, resulted in a horizontal shift of P(σ) curves to lower σ values. Some deviations from the Weibull distribution can be associated with the weakest fibers and is related to their damage during the sample preparation process. The Weibull parameters *m* and $\sigma_{0}$ of the reference and aged fibers are listed in Table 2.

**Figure 11:**
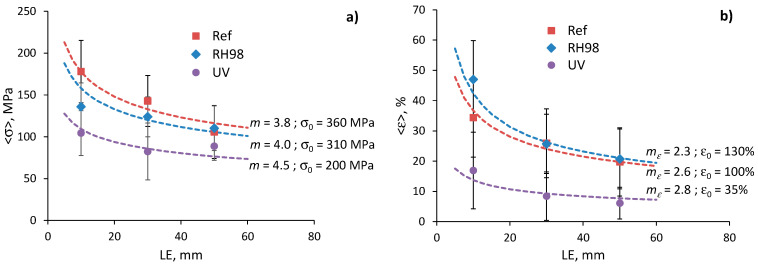
Strength (**a**) and failure strain (**b**) as functions of the gauge length of fibers from different environments. Solid lines are calculations by Equations (5) and (7), respectively; *α* = 1.

Figure 11a represents the dependence of the average strength on the gauge length of fibers and related predictions using Equation (5). The Weibull distribution parameters used for calculations are shown near the curves. These values are of the same order but different from those obtained by linear regression of the Weibull plots, and are listed in Table 2. Similar notes were made in other studies considering flax fiber strength distribution [21,26]. At the same time, changes in *σ*_0_ caused by fiber aging and determined by fitting Equation (5) correlate well with changes in the average strength (Section 4.2 and Table 2); the *σ*_0_ of RH98 and UV samples is 14% and 44% lower than that of the reference fibers with a *σ*_0_ = 360 MPa. The scale parameter *σ*_0_, related to the characteristic strength of the fibers, reduces due to the increased number of flaws caused by aging effects and fiber degradation.

The Weibull shape parameter, *m*, of the reference wool fibers takes the values from 3.6 to 5.6 (Table 2) that correlate well with data for various natural fibers reported elsewhere [21,23,25,33]. Weak correlations between the parameter *m* and the gauge length of fibers were established within each group of samples, although the literature data often reveal that *m* decreases as the fiber length increases, i.e., longer gauge length results in greater strength scatter [23,25,30]. An increasing trend is noticed for *m* determined by fitting the average strength data by Equation (5). In this formulation and data presentation, according to Figure 11a, a higher *m* is associated with a lower dependence, <*σ*>, vs. LE. Thus, aging, particularly UV irradiation, resulted in the mitigation of the gauge length effect on the strength of the wool fibers. This fact indicates a leveling of the number of defects per fiber length after their exposure to harsh environments.

### 4.4. Weibull Failure Strain Distribution Analysis

**Figure 12:**
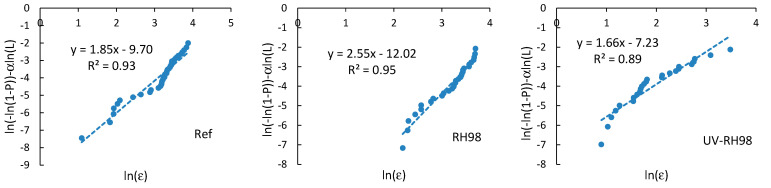
Weibull plots for failure strain of fibers in the reference and aged states at LE = 30 mm.

Failure strain distribution analysis is similar to that done for the strength and described in Section 4.3. The modified Weibull plots ln(−ln(1−P))−αln(L) vs. lnε for the reference and aged fibers (RH98 and UV-RH98) of a 30 mm gauge length are shown in Figure 12. Similar plots were obtained for all groups of fibers. Weibull parameters $m_{\varepsilon }$ and $\varepsilon_{0}$ according to Equation (6) are listed in Table 2. Due to weak correlations between the ultimate properties and diameter of the fibers, as mentioned above, it is assumed α=1.

**Figure 13:**
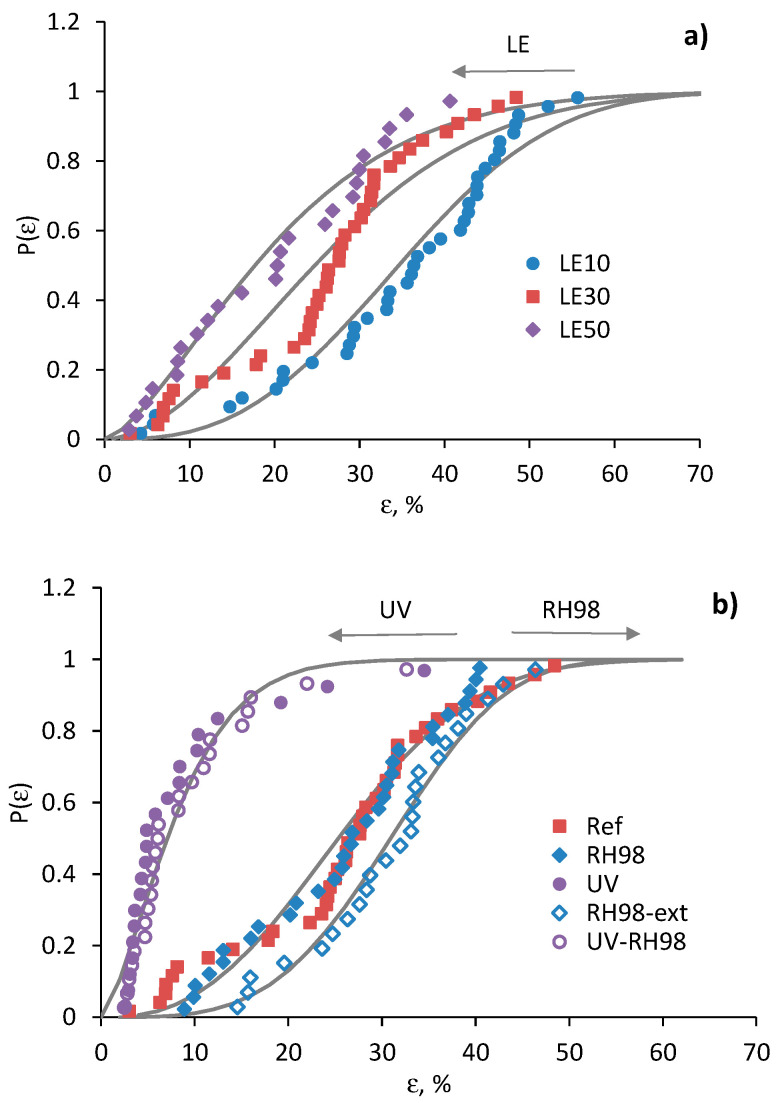
Failure strain distribution of the reference fibers of different gauge lengths (**a**) and fibers from different environments with an LE = 30 mm (**b**). Lines are calculations by Equation (5).

Figure 13 demonstrates the failure strain distribution of the reference and aged fibers. Overall, the data confirm the applicability of the two-parameter Weibull distribution for the failure strain analysis of wool fibers. However, the linear regression of the failure strain is characterized by lower correlation coefficients (*R*^2^ = 77–95%) compared to those obtained at the strength analysis (Table 2). $m_{\varepsilon }$ values are in the range of 1.3–4.6, which are also noticeably lower than *m*. These facts indicate a higher data dispersion for the failure strain compared to the strength, which can also be noticed by comparing the fitting data in Figure 10 and Figure 13. Similar to the strength data, an increase in the gauge length of fibers resulted in a horizontal shift of P(ε) curves to lower ε values (Figure 10a). The same effect is observed for UV-aged fibers and the opposite shift to a higher ε for moistened fibers.

The average failure strain dependencies on the gauge length of the fibers for the reference and aged fibers are shown in Figure 11b. The data are finely fitted by Equation (7), with parameters shown near the curves. The characteristic failure strain, $\varepsilon_{0}$, of the UV-aged fibers has the lowest value, 35%, which is 65% lower than that for the reference fibers. Due to plasticized effect of moisture, RH98 fibers possess higher deformations that appear in higher (up to 23%) $\varepsilon_{0}$ compared to the unaged counterparts. These results correlate well with changes in the average failure strain (Table 2). Analogously to the discussions in Figure 11a for the strength data, a higher $m_{\varepsilon }$ for UV-aged fibers is associated with a smoothened shape of the dependence <*ε*> vs. LE. In other words, the gauge length effect on the failure strain of the fibers is mitigated after UV exposure. An opposite trend is observed for the moisture-saturated fibers.

### 4.5. Optical Microscopy and SEM Investigations

**Figure 14:**
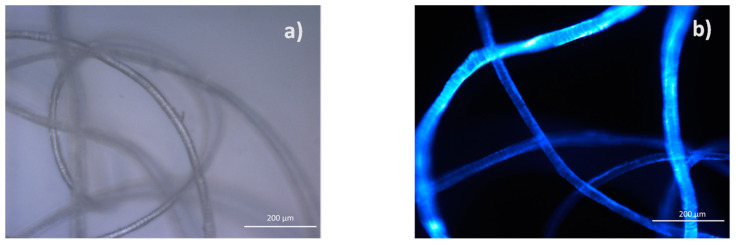
Optical micrographs of the reference wool fibers: original (**a**) and with a fluorescence filter set (**b**).

**Figure 15:**
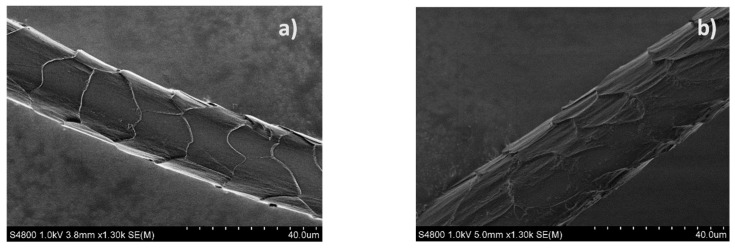
SEM images of the reference (**a**) and UV-aged wool fiber (**b**).

Optical micrographs of the reference wool fibers are shown in Figure 14. SEM pictures of the reference and UV-aged wool fibers are shown in Figure 15. Wool fibers are non-uniform and have a unique surface structure of overlapping scales called cuticle cells. A scaly surface of the fiber contributes to the wool’s ability to felt and enhance adhesion when used as a reinforcement in composites.

**Figure 16:**
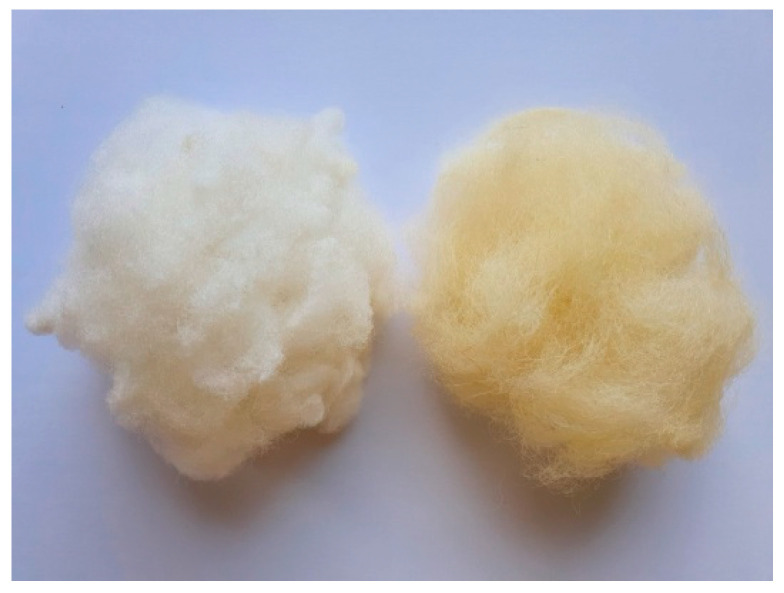
Photo of wool balls of the reference (**left**) and UV-aged (**right**) fibers.

Fibers conditioned under a humid environment did not show any noticeable changes in their visual appearance that could be observed by an optical microscope. SEM is not efficient for the analysis of surface changes of moisture-saturated wool fibers since moisture is desorbed during sample preparation and investigation, and it greatly affects the quality of images. UV irradiation damaged the wool fibers and appears on their loose and non-smooth surface. This resulted in the brittleness of the fibers. In addition, yellowing of UV-aged fibers was noticed. Although yellowing is hardly to be distinguished when considering single fibers, this is well seen by the eye for wool balls (Figure 16). Further investigations are needed for quantified color analysis of wool yellowing. Despite degradation effects, diameters of all fibers before and after aging remained within the distribution range (Section 4.1).

The wool cuticle is very resistant, which is due to the high degree of disulfide and isopeptide cross-linking. In the majority of cases, the amino acids in the cuticle are altered to a greater extent than in the cortex, as the outer layers of fibers receive more exposure to radiation. Since the cuticle protects the cortex, damage to this region usually occurs after extensive damage to the hair cuticle. These defects cause cystine degradation, but the exact mechanism is not precisely known [38].

The literature shows that photodegradation of cystine occurs via the C-S fission pathway and is unlike the chemical oxidation of cystine, which occurs mainly via the S-S fission pathway. Melanin provides a form of photochemical protection for hair proteins by absorbing and filtering falling radiation and then transferring this energy as heat. Its high absorption capacity can be attributed to an extensive system of conjugated carbonyl groups and double bonds. It not only traps a large part of the radiation but also immobilizes many of the free radicals, preventing the transfer of these free radicals into the keratin matrix. However, protecting the fiber proteins from light breaks down or bleaches the pigments. UV irradiation causes the formation of oxyradicals such as superoxide (O2^•−^) and hydroxyl (OH^•^). These compounds have a single unpaired electron in the outer orbital, which gives them a very strong ability to react, especially with molecules with a double bond structure, such as unsaturated lipids. These changes are believed to be caused by UV light-induced oxidation of sulfur-containing molecules in the fiber cortex [39].

In peptides and proteins, in particular, the hydrolytic reaction breaks the amide bond of the peptide and protein with a water molecule. This process results in the conversion of asparagine (Asn) to aspartic acid (Asp) (deamidation), the formation of protein fragments (peptide bond cleavage), or the cyclization of adjacent amino acid residues such as Arg-Pro and Lys-Pro [40]. Further investigations are needed to comprehend the degradation phenomena caused by environmental aging. Some results of the structural characterization of wool fibers will be highlighted in the next study.

## 5. Conclusions

Latvian dark-headed sheep wool fibers, those that are virgin, and those after environmental aging, were tested. The results of single fiber tensile tests showed a noticeable gauge length effect on the mechanical characteristics of the fibers. The strength and failure strain of long (50 mm) fibers are about 40% lower than those for short (10 mm) fibers. The elastic modulus decreased, but strength showed weak correlations with the growing diameter of fibers of each group. Environmental aging significantly affected the wool’s mechanical performance. Aged fibers possessed lower strength and stiffness compared to their pristine counterparts. Long-term conditioning of fibers under a humid environment resulted in a decrease of the strength and elastic modulus of 34% and 43%, respectively. UV-aged fibers possessed up to a twofold reduction in the ultimate mechanical characteristics. The most severe degradation effect was observed under the coupled influence of moisture and UV irradiation.

Weibull distribution was utilized for statistical data analysis. The strength and failure strain was estimated by the two-parameter Weibull model, and their gauge length dependences were well-fitted by the model. The dispersion of the results is compatible with the nature of fibers and allows characterization of its mechanical behavior with reasonable confidence. Moisture-saturated and UV-aged fibers are characterized by less extensive strength dependencies on the fiber length. Environmental aging resulted in a horizontal shift of the strength and failure strain distributions to lower values.

The obtained results can be useful for material designers and engineers in selecting appropriate eco-friendly components for specific applications and predicting their environmental durability. Comprehensive characterization of wool fibers will promote their use in novel technical applications, contributing in this way to the effective use of local natural resources and waste management.

## Figures and Tables

**Table 1:** Details on fiber conditioning under different environments.

Notation	Environment	Duration	Gauge Length (LE), mm
Ref	Ambient: RH = 37 ± 5%, *T* = 22 ± 2 °C	-	10, 30, 50
RH98	RH = 98%, *T* = 22 ± 2 °C	1 week	10, 30, 50
RH98-ext	RH = 98%, *T* = 22 ± 2 °C	2 months	30
UV	*I* = 4 mW/cm^2^, *T* = 35 ± 2 °C; “Ref” fibers	24 h	10, 30, 50
UV-RH98	*I* = 4 mW/cm^2^, *T* = 35 ± 2 °C; “RH98” fibers	24 h	30

**Table 2:** Mechanical characteristics and Weibull distribution parameters of the wool fibers.

Environment	LE, mm	Number of Tests	<*d*> ^1^, μm	<*E*>, GPa	<*σ*>, MPa	<*ε*>, %	*m*	*σ*_0_ (*R*^2^, %) ^2^	$m_{\varepsilon }$	$\varepsilon_{0}$ (*R*^2^, %)
Ref	10	20	37.63 (±4.50)	4.58 (±1.13)	178.0 (±37.2)	34.4 (±13.1)	4.80	311.6 (96%)	2.77	90.6 (94%)
	30	42	38.14 (±5.72)	3.93 (±0.61)	142.8 (±30.3)	25.9 (±11.4)	5.62	283.1 (91%)	1.85	190.4 (93%)
	50	25	37.14 (±4.66)	4.16 (±0.61)	105.8 (±31.7)	19.7 (±11.3)	3.59	353.43 (98%)	1.47	324.5 (96%)
RH98	10	25	37.29 (±4.55)	2.31 (±0.51)	136.0 (±28.3)	47.0 (±12.8)	5.33	226.9 (98%)	4.57	86.4 (95%)
	30	30	41.68 (±5.39)	3.06 (±0.59)	124.1 (±23.8)	25.7 (±9.8)	5.99	236.2 (94%)	2.55	111.0 (95%)
	50	25	36.97 (±3.86)	2.84 (±0.51)	110.3 (±26.7)	20.7 (±9.9)	4.65	279.3 (96%)	1.88	191.6 (95%)
RH98-ext	30	24	39.42 (±5.52)	2.23 (±0.53)	94.4 (±28.3)	30.6 (±8.6)	3.73	259.9 (98%)	3.66	86.6 (97%)
UV	10	26	35.97 (±3.80)	3.24 (±0.58)	104.5 (±26.8)	16.7 (±12.7)	3.95	207.1 (95%)	1.32	103.2 (94%)
	30	23	40.05 (±8.10)	3.95 (±1.23)	82.4 (±33.9)	8.4 (±8.0)	2.56	353.2 (98%)	1.45	94.8 (79%)
	50	25	34.83 (±5.00)	4.02 (±0.66)	88.8 (±16.9)	6.1 (±5.2)	6.06	182.2 (95%)	1.72	65.8 (77%)
UV-RH98	30	26	40.09 (±5.89)	2.95 (±0.76)	66.6 (±22.1)	9.0 (±7.0)	3.05	228.7 (98%)	1.66	77.9 (89%)

^1^ The mean diameter is the average diameter of *N* fibers, while the diameter for each fiber is the average value from 5 measuring points along the fiber length. ^2^ *R*^2^ is the correlation coefficient in the Weibull plots.

## Data Availability

Not applicable.
